# Lipoproteins/peptides are sepsis-inducing toxins from bacteria that can be neutralized by synthetic anti-endotoxin peptides

**DOI:** 10.1038/srep14292

**Published:** 2015-09-22

**Authors:** Guillermo Martinez de Tejada, Lena Heinbockel, Raquel Ferrer-Espada, Holger Heine, Christian Alexander, Sergio Bárcena-Varela, Torsten Goldmann, Wilmar Correa, Karl-Heinz Wiesmüller, Nicolas Gisch, Susana Sánchez-Gómez, Satoshi Fukuoka, Tobias Schürholz, Thomas Gutsmann, Klaus Brandenburg

**Affiliations:** 1 Universidad de Navarra, Dept. de Microbiología y Parasitología, Irunlarrea 1, 31008 Pamplona, Spain; 2 Divisions of Biophysics, Research Center Borstel, Leibniz-Center for Medicine and Biosciences, Parkallee 1-40, 23845 Borstel, Germany; 3 Innate Immunity, Research Center Borstel, Leibniz-Center for Medicine and Biosciences, Parkallee 1-40, 23845 Borstel, Germany; 4 Cellular Microbiology, Research Center Borstel, Leibniz-Center for Medicine and Biosciences, Parkallee 1-40, 23845 Borstel, Germany; 5 Bioanalytical Chemistry, Research Center Borstel, Leibniz-Center for Medicine and Biosciences, Parkallee 1-40, 23845 Borstel, Germany; 6 Clinical and Experimental Pathology, Research Center Borstel, Leibniz-Center for Medicine and Biosciences, Parkallee 1-40, 23845 Borstel, Germany; 7 EMC microcollections, Sindelfinger Straße 3, 72070 Tübingen, Germany; 8 National Institute of Advanced Industrial Science and Technology AIST, Takamatsu, Japan; 9 Universitätsklinikum Aachen, Department of Intensive Care and Intermediate Care, Pauwelsstr. 30, 52074 Aachen, Germany

## Abstract

Sepsis, a life-threatening syndrome with increasing incidence worldwide, is triggered by an overwhelming inflammation induced by microbial toxins released into the bloodstream during infection. A well-known sepsis-inducing factor is the membrane constituent of Gram-negative bacteria, lipopolysaccharide (LPS), signalling via Toll-like receptor-4. Although sepsis is caused in more than 50% cases by Gram-positive and mycoplasma cells, the causative compounds are still poorly described. In contradicting investigations lipoproteins/-peptides (LP), lipoteichoic acids (LTA), and peptidoglycans (PGN), were made responsible for eliciting this pathology. Here, we used human mononuclear cells from healthy donors to determine the cytokine-inducing activity of various LPs from different bacterial origin, synthetic and natural, and compared their activity with that of natural LTA and PGN. We demonstrate that LP are the most potent non-LPS pro-inflammatory toxins of the bacterial cell walls, signalling via Toll-like receptor-2, not only *in vitro*, but also when inoculated into mice: A synthetic LP caused sepsis-related pathological symptoms in a dose-response manner. Additionally, these mice produced pro-inflammatory cytokines characteristic of a septic reaction. Importantly, the recently designed polypeptide Aspidasept^®^ which has been proven to efficiently neutralize LPS *in vivo*, inhibited cytokines induced by the various non-LPS compounds protecting animals from the pro-inflammatory activity of synthetic LP.

Severe infectious diseases are still a major threat to human health, especially in the light of the global proliferation of antibiotic resistant pathogens. To make this situation worse, the development of new antibiotics stagnated during the last decades^1^. Complicated infections cause with increasing frequency a severe pathology called sepsis^2^, which is the leading cause of death in critical care units^3^,^4^. Every year, sepsis strikes more than a million Americans and it is estimated that 28–50% of them die—far more than the number of U.S. deaths from prostate cancer, breast cancer and AIDS combined^5^,^6^. The majority of sepsis cases is caused by a disproportionate immune response to bacterial products, mainly LPS in the case of Gram-negative bacteria^7^. LPS forms the outer leaflet of the outer membrane for nearly all Gram-negatives. Besides LPS, also other amphiphilic molecules, i.e., lipopeptides and lipoproteins (LP) are found in bacterial envelopes, in Gram-negative as well as in Gram-positive bacteria and in mycoplasma^8^. Recent studies provided substantial evidence that cell-envelope LP from Gram-positive and Gram-negative bacteria trigger inflammatory responses by activation of Toll-like receptor TLR2^9^,^10^ at minute concentrations and thus seem to be important sepsis-inducing factors beyond LPS. In some prior publications also Gram-positive lipoteichoic acids (LTA) as well as peptidoglycans (PGN) from the cell envelope of Gram-positive organisms were described exhibiting significant cytokine induction via TLR2 in human mononuclear cells (MNC)^11^. Observations that purified or synthetic LTA or PGN were devoid of such activity likely indicates that previous preparations could be contaminated with LP and that this compound may be responsible for their TLR2 activating properties^12^, (review in^13^).

A major contribution to clarify the immunostimulatory potency of LP involved the construction of a ∆*lgt*-mutant of *S. aureus* strain SA113, which was deficient in the lipidation of the pre-LP and showed attenuation in immune activation and growth^14^. Subsequent studies demonstrated that the LP are the predominant TLR2-stimuli in LTA preparations of *S. aureus* and not the LTA itself^12^,^15^. Furthermore, studies using purified LTA from *lgt*-mutants^15^ as well as synthetic LTA^16^, (reviewed by Schmidt *et al.*^17^) or PGN part structures^18^ did not show any TLR2-mediated activity. This indicated again a possible LP contamination in the previously used LTA and PGN preparations responsible for the originally determined bioactivity, thus proving that TLR2 is most likely a specific receptor for LP^13^. The mechanism of this immune activation in humans was determined at the atomic level in several X-ray diffraction studies, namely the signaling induced by triacylated LP occurs via a TLR2/TLR1-heterodimer (as shown by analyzing a hTLR2-hTLR1-Pam_3_CSK_4_ co-crystal)^19^, whereas diacylated LP signals via a TLR2/TLR6-heterodimer (demonstrated by solving a mTLR2-mTLR6-Pam_2_CSK_4_ co-crystal)^20^. In murine cells LP that signal via both TLR2/TLR1 and TLR2/TLR6 heterodimer, have been described^21^. In addition, it was shown, that TLR2 is indeed able to bind LTA, but that this binding induces levels of heterodimer formation below those necessary to trigger an intracellular signaling process^20^. Despite these findings there are recent works that still favour the notion that LTA are the Gram-positive counterparts of LPS^11^.

To contribute to settle this issue, we first studied the pro-inflammatory activity of selected non-LPS-related LP structures of different natural and synthetic origin. All these compounds are exposed on the microbial cell surface and could bear pathogen-associated molecular patterns (PAMPs) recognizable by innate immune cells receptors. Furthermore, we studied whether synthetic anti-LPS peptides (SALPs) that efficiently neutralized both LPS and bacterial whole cells^22^,^23^,^24^ could interact with LP as well as with LTA and PGN. The *in vitro* and *in vivo* data gathered in the present study reveal that, in the absence of LPS, LP triggers severe inflammatory host responses with a potency similar to that of LPS. Interestingly, our results show that all the non-LPS amphiphiles tested can be efficiently neutralized by SALPs suggesting that these compounds could form the basis to develop broad-spectrum anti-sepsis drugs. For this, however, some further investigations are necessary outside the scope of this paper regarding the application of the SALP in more detailed models of Gram-positive bacteremia.

## Results

In this section, we first present *in vitro* data on the production of pro-inflammatory cytokines by human cells in response to LP or LTA from different origin and its neutralization by SALP. Then, we used a mouse model of acute toxemia to characterize the *in vivo* expression of those cytokines and to study if SALP can protect animals against lethal LP-induced sepsis. It is worth noting that in preceding studies we demonstrated that Aspidasept^®^ (Pep19-2.5) efficiently inhibits the LPS-induced cytokine expression in human MNCs even at [LPS]:[Aspidasept^®^] weight ratios as low as 1:1^24^,^25^,^26^.

### Staphylococcal LP

**Figure 1:**
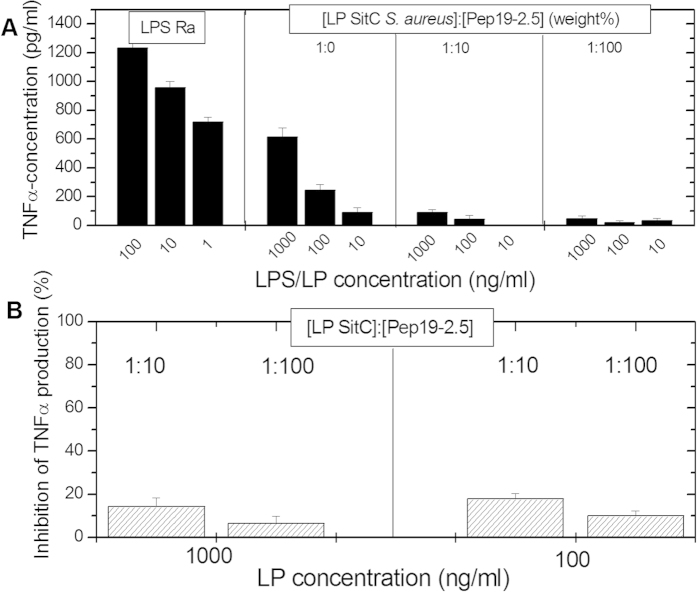
(**A**) Production of tumor-necrosis-factor α in human macrophages induced by LPS from *Salmonella minnesota* R60, which has a complete core oligosaccharide and corresponds to the bioactive part of wild type LPS, and by the lipoprotein SitC from *Staphylococcus aureus*, each at three different concentrations, the latter also in the presence of different concentrations of Aspidasept^®^ 1:10 and 1:100 [LP]:[Pep] weight%. (**B**) Presented is the inhibition of the TNFα production at the two excess concentrations (10:1 and 100:1 weight %). Depicted are the means ± SEM of 3 independent experiments.

In Fig. 1A the stimulation of TNFα production in human macrophages caused by incubation with a native LP (SitC from *Staphylococcus aureus*) is compared to the equivalent stimulation by LPS Ra, a LPS with a complete core oligosaccharide, from *Salmonella minnesota*. This LPS was taken because it corresponds to the bioactive fraction within wild-type LPS. Two key observations are made: (i), LPS was at least 10 times more potent than LP at inducing TNFα expression in macrophages; and (ii), the addition of Aspidasept^®^ almost totally abrogated LP-dependent TNFα secretion at all tested concentrations. In Fig. 1B, the inhibition efficiency of Pep19-2.5 is summarized for the two LP concentrations 1000 and 100 ng/ml from three independent measurements. The data show clearly, that already a 10:1 excess of peptide is sufficient for a strong inhibition.

### LP from spirochetes and Gram negative pathogens

**Figure 2:**
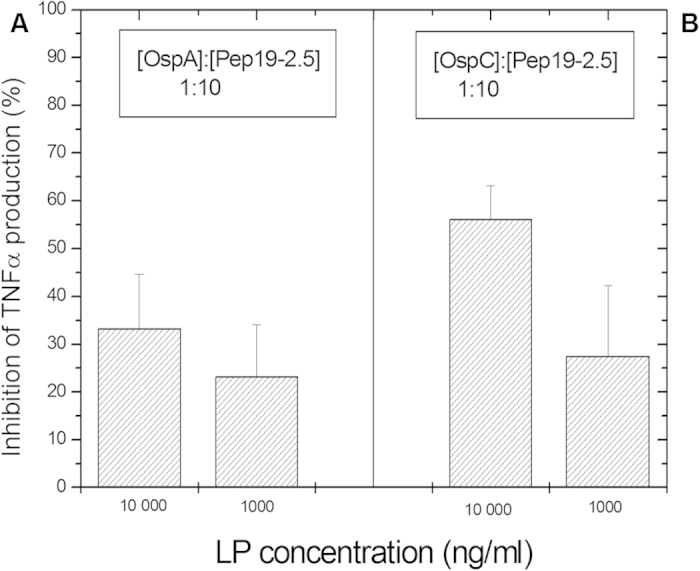
Inhibition of the production of tumor-necrosis-factor α in human mononuclear cells induced by synthetic lipopeptide homologues ? to the surface antigens of *Borrelia burgdorferi* OspA (A) and OspC (B) at a concentration of 10000 and 1000 ng/ml in the absence and presence of a 10:1 weight % excess concentration of Aspidasept^®^. Depicted are the means ± SEM of 3 independent experiments.

Using the same *in vitro* system, we determined the TNFα stimulating potential of synthetic LP identical to the N-terminal part of the surface antigens from *Borrelia burgdorferi* OspA and OspC and from the outer membrane proteins P6 of *Haemophilus influenzae* and H8 of *Neisseria gonorrhoeae*. For the *Borrelia* LP OspA and OspC, our results demonstrate that concentrations up to 1000 to 10000 ng/ml were necessary to induce TNFα levels similar to those brought about by the lowest amount of LPS tested in the previous assay (at 1000 to 10 000 ng/ml OspA and C, 500 to 1000 pg/ml TNFα are produced by the MNCs). Notably, Aspidasept^®^ inhibited the LP dependent cytokine production when added at a peptide:LP ratio of 10:1 (Fig. 2) and brought it down to undetectable levels at a 100:1 concentration ratio (data not shown). For the *Haemophilus* and *Neisseria* LP, also a concentration of 10 μg/ml was necessary to induce an observable TNFα secretion, and there was no detectable amounts of this cytokine when adding 100 ng/ml of either LP (data not shown).

### Mycoplasma LP

**Figure 3:**
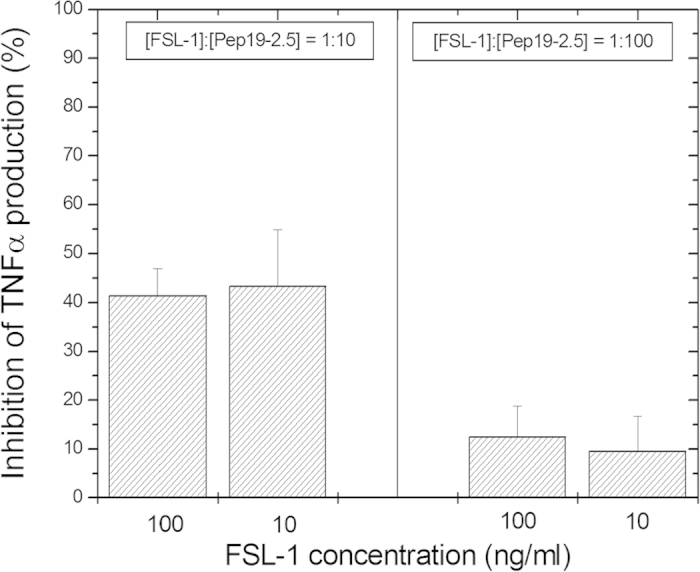
Inhibition of the production of tumor-necrosis-factor α in human mononuclear cells induced by synthetic lipopeptide FSL-1 (fibroblast-stimulating lipopeptide), the N-terminal part of the lipoprotein from *Mycoplasma salivarium* at a concentration of 100 and 10 ng/ml in the absence and presence of an excess concentration of Aspidasept^®^ 10:1 weight%. Depicted are the means ± SEM of 3 independent experiments.

The pro-inflammatory activity of a bioactive compound from *Mycoplasma*, the LP FSL-1 (fibroblast-stimulating LP) was also investigated. Remarkably, FSL-1 LP stimulated the MNC with potency similar to that of LPS. Addition of a 10-fold excess of Aspidasept^®^ with respect to LP led already to a considerable reduction of the TNFα secretion, and at a 100:1 excess the cytokine production was nearly completely abolished (Fig. 3). Similar results were obtained with the compound MALP-2 (macrophage-activating LP), whose pro-inflammatory activity was also subject to nearly complete inhibition by Aspidasept^®^ at a 100:1 weight% excess of the peptide (data not shown).

### LTA and PGN

**Figure 4:**
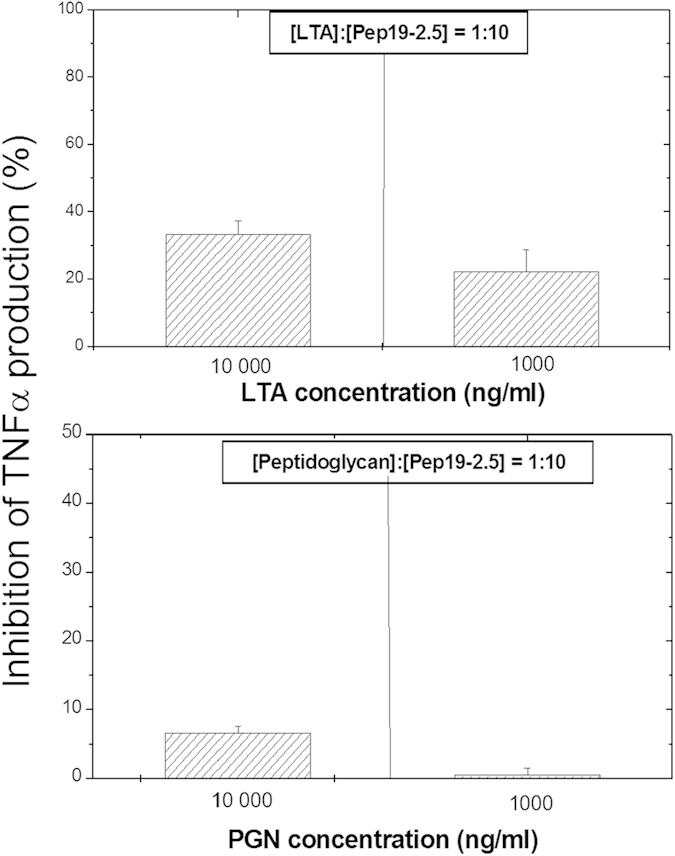
Inhibition of the production of tumor-necrosis-factor α in human mononuclear cells induced by purified lipoteichoic acids (LTA) or by peptidoglycan (PGN) both isolated from *S. aureus* SA113Δ*lgt*, each at 10000 and 1000 ng/ml in the absence and presence of Aspidasept^®^ at 10:1 excess weight%. Depicted are the means ± SEM of 3 independent experiments.

In exemplary measurements we have also analysed two commercial lipoteichoic acid (LTA) preparations from *Bacillus subtilis* and *Staphylococcus aureus*. Cytokine data for the two LTA in the absence and presence of Pep19-2.5 and in comparison to LPS show that LTA from *B. subtilis* exhibits some TNFα induction, although still one order of magnitude lower than LPS, and a complete inhibition by Pep19-2.5 was already noticeable at a 10:1 weight% excess (see also^27^). The cytokine induction activity of the other commercial preparation LTA from *S. aureus* was in contrast much lower, and was also inhibited when the peptide was added (data not shown). To study whether LTA and PGN had TNFα stimulating activity by themselves these two compounds were isolated from *S. aureus* SA113Δlgt, a mutant deficient in the lipidation of LP precursors. The LTA and PGN preparations showed a low pro-inflammatory activity in the range 200 to 250 pg/ml even when added at 10.000 ng/mL (10 μg/ml). Irrespective of this fact, addition of a 10-fold excess of Aspidasept^®^ (Fig. 4) effectively reduced the LTA-dependent TNFα response and totally inhibited the equivalent response induced by PGN.

### Shortened triacylated LP

**Figure 5:**
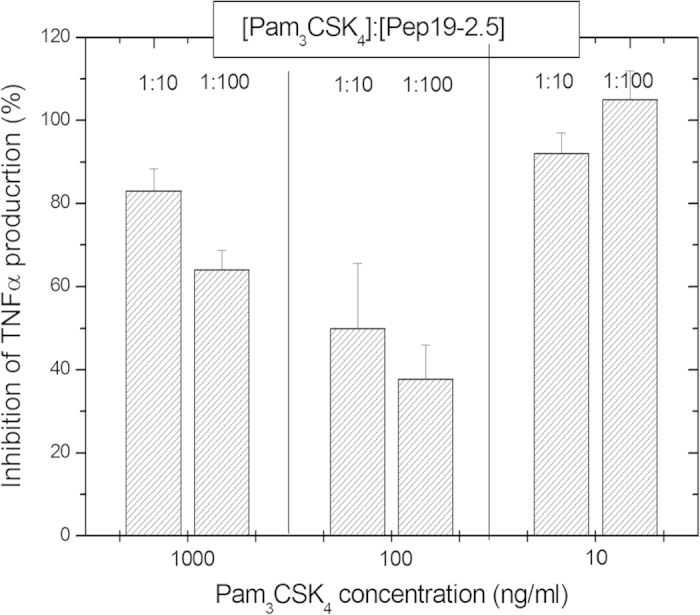
Inhibition of the production of tumor-necrosis-factor α in human mononuclear cells by the synthetic lipid anchored partial structure Pam_3_CSK_4_ of bacterial lipoproteins at 1000, 100, and 10 ng/ml concentrations in the absence and presence of an excess concentration of Aspidasept^®^ 10:1 and 100:1 weight %. Depicted are the means ± SEM of 3 independent experiments.

As shortened variant of triacylated LP, synthetic compound Pam_3_CSK_4_ at a concentration of 100 ng/ml was tested in the absence and presence of excess Aspidasept^®^ 100:1 weight%. The data demonstrated that Pam_3_CSK_4_ is also able to stimulate MNC although at a markedly lower level than LPS. Also at 10 ng/ml some activation takes place, but not at 1 ng/ml (data not shown). As shown in Fig. 5, the addition of compound Aspidasept^®^ had only a minor inhibitory effect on cytokine production, even at high concentrations [LP]:[Pep] 1:100, in particular at the lowest Pam_3_CSK_4_ concentration of 10 ng/ml. This means that the reduction of the length of the amino acid sequence in the polar moiety of the LP, as compared to the other LP compounds investigated above, apparently leads to less binding of the peptide to the LP (see the ITC experiments shown below).

### Test of transfected HEK cells

**Figure 6:**
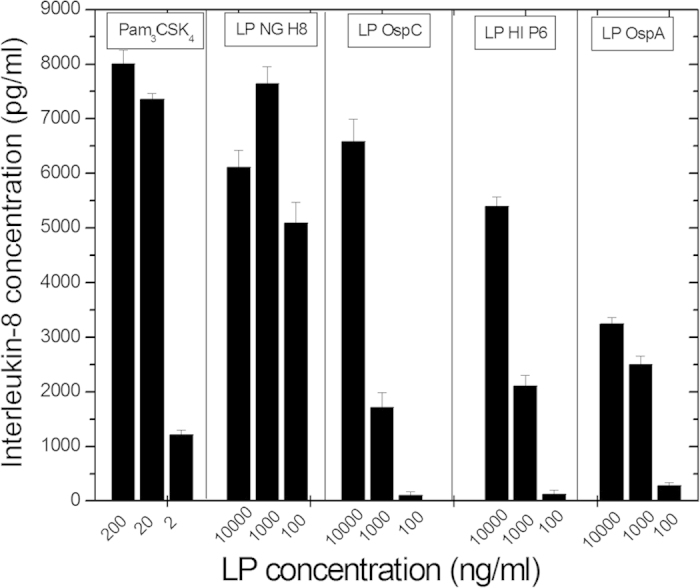
Production of the chemokine interleukin-8 in a HEK cell system transfected with Toll-like-receptor TLR2 by Pam_3_CSK_4_, LP from *H. influenza* (HI P6), from *N. gonorr* (NG H8) and from *B. burgdorferi* OspA and OspC. Presented is one exemplary experiment out of two. Errors bars are from the determination of TNFα in duplicate.

The ability of selected LP, i.e., LP Osp A and OspC, LP H8 and P6, to stimulate interleukin-8 (IL-8) production via TLR2 was quantified in HEK cells transfected with a TLR2 expression vector. In independent experiments, LPS was used as control of TLR4-dependent signaling in HEK cells transfected with a TLR4 expression vector. As expected, TLR4-transfected cells expressed high levels of IL-8 in response to LPS, and nearly no IL-8 signal for the four LP except for LP HI P6, which induced significant amounts of IL-8 when added at high concentration (10 μg/ml). In TLR-2 transfected cells, all 4 LP as well as Pam_3_CSK_4_ exhibited considerable IL-8 secreting activity, at relatively high LP concentrations (Fig. 6), which for the LP P6 and H8 is significantly higher as compared to the TNFα levels reached in previous experiments with human MNC. It should be noted at this place, however, that primary cells (MNCs) and cell lines (HEK) are quantitatively not necessarily comparable.

### *In vivo* stimulation in mice

In preliminary assays, Balb/C mice were used for the analysis of the global cytokine response stimulated by the intraperitoneal inoculation of 20 μg/animal of either LPS or LP SitC. Cytokine quantification in serum was performed at 90 min and 4 h after injection and included TNFα, interferon-γ (IFNγ), interleukin-6 (IL-6), and IL-12 (p40). These assays demonstrated that LPS induced a very potent and uniform stimulation of all those cytokines, in particular at 4 h post-inoculation, whereas the response to LP was significantly lower. The cytokine IL-6 was stimulated by both compounds to a higher level than the other cytokines (data not shown).

**Figure 7:**
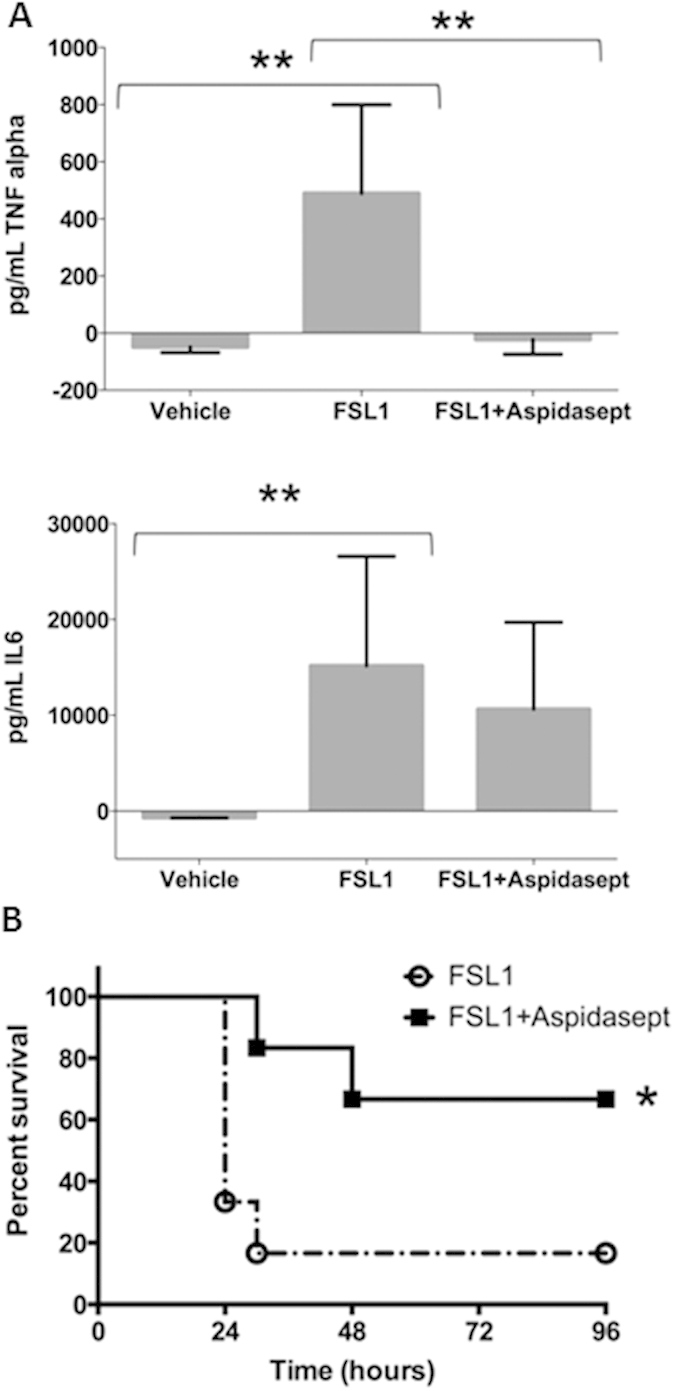
(**A**) Serum levels of tumor-necrosis-factor α (upper panel), and interleukin-6 (lower panel) in a group of mice (n = 4) intraperitoneally (i.p.) inoculated with 40 μg of FSL-1 (central bars) compared to another group receiving i.p. 400 μg of Aspidasept^®^ immediately after an identical FSL-1 challenge (right bars; n = 5) and a third group administered only with pyrogen free saline i.p. (vehicle; left bars; n = 5). TNFα (top) and IL-6 (bottom) levels were measured at 1.5 h or 4 h after challenge, respectively, coinciding with the peak of cytokine secretion, as determined in preliminary experiments. Double asterisks denote significant statistical differences between the two groups indicated by the bracket (p < 0.01; Mann-Whitney U test). (**B**) Survival rate of Balb/C mice intraperitoneally inoculated with FSL-1 (open circles; n = 6) or receiving FSL-1 and then treated with Aspidasept^®^ (solid squares; n = 6). All the animals received FSL-1 (4 μg/mouse) co-administered with D-galactosamine (18 mg/mouse) intraperitoneally. A group of animals was treated with Aspidasept^®^ (200 μg/mouse) immediately after FSL-1 challenge at a different site of the peritoneum and animal mortality was monitored every 4 h for 4 days. Results were globally analysed by means of a Kaplan-Meier survival analysis. Asterisk denotes significant statistical differences (p < 0.05).

In a second set of experiments, pro-inflammatory cytokine levels (TNFα and IL-6) were determined in mice 90 min and 4 hours after the intraperitoneal inoculation of 40 μg of FSL-1. A duplicate group of animals received 400 μg of Aspidasept^®^ immediately after FSL-1 challenge at a different site of the peritoneum. As shown in Fig. 7A, FSL-1 induced a potent TNFα (top) and IL-6 (bottom) response in the animals. Whereas levels of the former cytokine peaked at 90 min, the latter peaked at 4 hours (data not shown). Remarkably, treatment with Aspidasept^®^ totally inhibited FSL-1 induced TNFα stimulation in mice but only caused a slight reduction in IL-6 levels.

In a third set of experiments, galactosamine-sensitized mice, which is known to considerably increase the sensitivity of mice to bacterial toxins, were inoculated with increasing concentrations of FSL-1 and animals were monitored for symptoms of toxemia during 48 h. As shown in Table 1, this assay clearly demonstrated a dose-response relationship between the amount of the LP given to the animals and the severity of the symptoms displayed by them at all time-points after FSL-1 challenge. Interestingly, the amount of FSL-1 necessary to kill 100% of the animals in 48 h (LD_100_) as determined in this experiment, 500 ng/mouse (Table 1), was only 10 times higher than the equivalent LD_100_ of *Salmonella minnesota* LPS when tested under identical experimental conditions^24^.

To evaluate a possible therapeutic effect of Aspidasept^®^ on toxemia caused by FSL-1, galactosamine-sensitized animals received by the intraperitonal route first 4 μg of the LP and immediately afterwards 200 μg of Aspidasept^®^ at a different site of the peritoneum (i.e., the peptide excess concentration was 50:1 w/w). Animals inoculated with FSL-1 displayed symptoms of sepsis (reduced motor activity, lethargy, shivering, and piloerection) within the first 120 min. At 12 h post-inoculation, these symptoms worsened and areas of clotted blood appeared in the nails, a pathology characteristic of septic shock. Administration of Aspidasept^®^ resulted in a marked reduction of symptoms in 80% of the animals. The data presented in Fig. 7B clearly show a significant survival benefit of the mice treated with Aspidasept^®^, which continued for up to 4 days.

### Isothermal calorimetric titration of LP with Aspidasept^®^

Isothermal titration calorimetry (ITC) allowed us to characterize thermodynamically the LP-Aspidasept^®^ interaction. For this assay, FSL-1 and Pam_3_CSK_4_ were selected, since they displayed a disparate behavior when interacting with Aspidasept^®^, with good degree of inhibition by the peptide of the former (Fig. 3) and no significant influence when added to the latter (Fig. 5).

**Figure 8:**
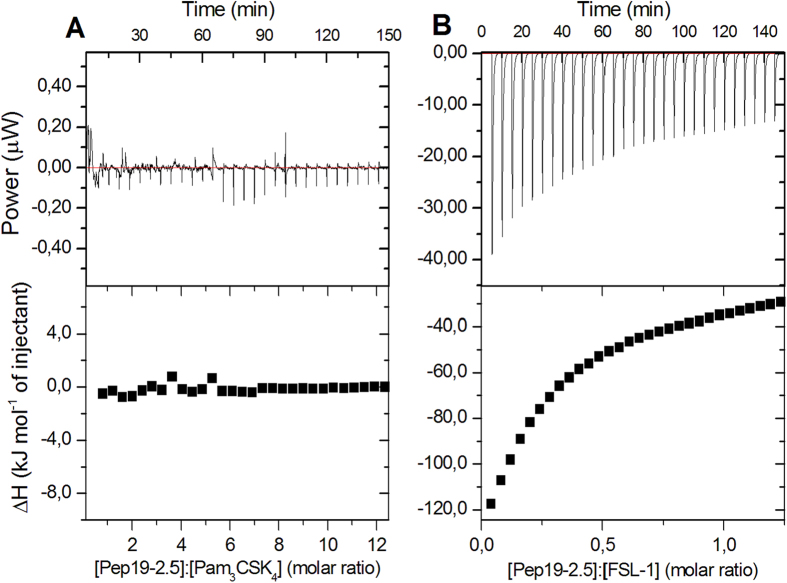
Isothermic calorimetric experiments of the titration of Aspidasept^®^ to the lipopeptide Pam_3_CSK_4_ (A) and FSL-1 (B). The lipopeptides were prepared as 0.05 mM dispersions and the peptide at a concentration of 1 mM was titrated in 3 μl portions every 5 min at 37 °C.

The analysis of the Pam_3_CSK_4_:peptide interaction revealed a weak exothermic reaction (Fig. 8A) lacking the typical binding kinetics, whereas the FSL-1:peptide interaction was indicative of an exothermic reaction with saturation kinetics (Fig. 8B). These data indicate that the inability of Aspidasept^®^ at low or medium concentrations to inhibit the Pam_3_CSK_4_ dependent cytokine secretion is the consequence of poor affinity of the peptide towards the LP.

### Förster resonance energy transfer of peptide binding to LP

**Figure 9:**
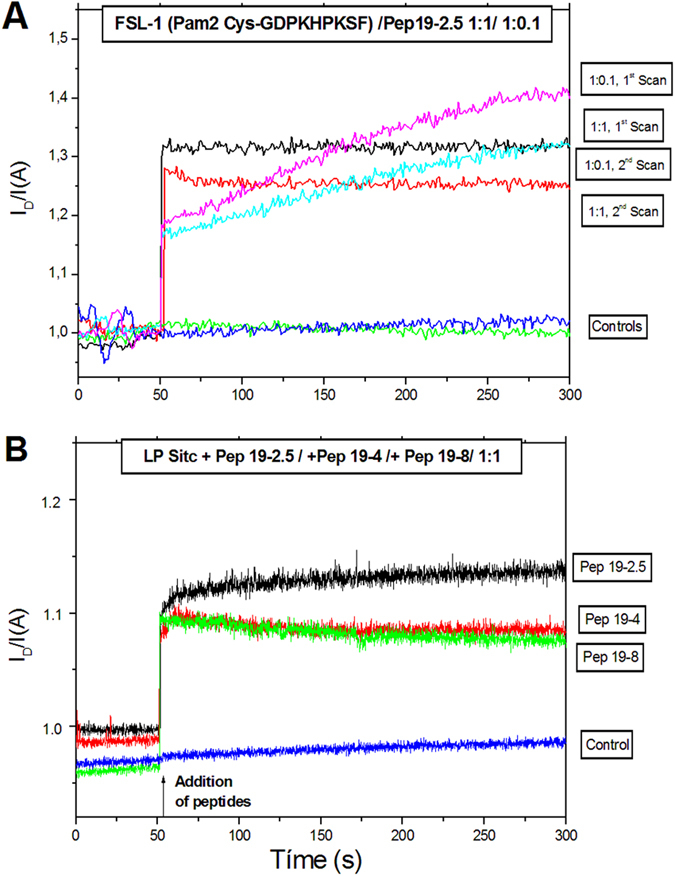
Förster resonance energy transfer (FRET) spectroscopic data of the intercalation of Aspidasept^®^ into FSL-1 aggregates (A) and of Pep19-4, Pep19-8, and Aspidasept^®^ into LP SitC aggregates (B). To the LP at a concentration of 1 μM the peptides were added after 50 s and the concentration ratio I_D_/I_A_ was monitored for 300 s. ad A) FSL-1:Pep19-2.5 1:0.1 M/M: 1^st^ scan: magenta, 2^nd^ scan: light blue. FSL-1:Pep19-2.5 1:1 M/M: 1^st^ scan: black, 2^nd^ scan: red. Controls (only FSL-1): blue/green ad B) LP SitC: Pep19-2.5 1:1 M/M: black. LP SitC: Pep19-4 1:1 M/M: red. LP SitC: Pep19-8 1:1 M/M: green. Control (only LP SitC) blue.

To gather more information about the LP-Aspidasept^®^ interaction at the molecular level, we studied whether Aspidasept^®^ could intercalate into LP aggregates by FRET. In these assays, we investigated two LP systems, namely the FSL-1 sample in the presence of different concentrations of Aspidasept^®^ (Fig. 9A), and the LP SitC from *S. aureus* in the presence of three peptides, Aspidasept^®^, Pep19–4 and Pep19–8 (Fig. 9B). As depicted in Fig. 9A, the addition of Aspidasept^®^ to FSL-1 led to an instantaneous incorporation of the former into the LP aggregate, already at the low peptide concentration ([LP]:[Aspidasept^®^] 1:0.1 molar). The increase in peptide concentration did not lead to a further increase in intercalation, apparently due to saturation.

The data on the incorporation of three different peptides into LP SitC (Fig. 9B) show very similar instantaneous and strong increases of the FRET signals, indicating a general tendency of the three peptides to efficiently intercalate into LP aggregates independently of their inhibition efficiency in the biological experiments [Pep19–4 and Pep19–8 have only moderate or low inhibition efficiency, respectively, see^25^].

## Discussion

In the present study the role of essential pathogenicity factors of bacteria, which are responsible for the severe inflammation reactions in mammals but are structurally non-related to endotoxin, were investigated. In previous publications a variety of different amphiphilic membrane-associated compounds were described exerting manifold immunologically relevant reactions, but no clear line has been drawn so far. Lipoteichoic acids and peptidoglycans as well as lipopeptides and lipoproteins all were found or suspected to be inflammation-inducing toxins (pathogenicity factors) from Gram-positive bacteria, mycoplasma and non-LPS related Gram-negative bacterial components^8^,^11^,^28^,^29^. In particular, no reports are known to the best of our knowledge that conclusively identify the non-endotoxin related sepsis-inducing compounds from bacteria including mycoplasma species.

The use of the various shortened lipopeptides used here instead of the parental lipoproteins should be justified by the fact that the stimulation property of a given lipoprotein depends exclusively on the presence and structure of its lipid moiety, as proven exemplarily for the shortened variant Pam_3_CSK_4_, similar as in the case of LPS in which the lipid A moiety and not the sugar part is the ‘endotoxic principle’^7^,^9^,^10^,^22^.

As a second essential purpose of this work, the ability of previously described antimicrobial peptides^24^,^25^, called synthetic anti-LPS peptides (SALP) to inhibit inflammation reactions due to various LP, LTA and PGN, from natural and synthetic origin, was investigated. The focus here was on the anti-septic therapeutic drug candidate Aspidasept^®^.

### Cytokine induction

The data reveal that all compounds investigated induce cytokine production in human MNC, measured as TNFα expression, but with considerable variations in their potency. The investigated lipopeptides/-proteins, i.e., lipoprotein from *S. aureus* SitC (Fig. 1), LP OspA and OspC from *B. burgdorferi* (Fig. 2), LP from *H. influenzae* and *N. gonorroeae* and staphylococcal LTA (Fig. 4) induce cytokines only at a 10- to 1000-fold higher concentration than LPS (Fig. 1). Staphylococcal PGN exhibits only negligible inflammatory activity even at high concentrations (Fig. 4). In mouse experiments (Fig. 7) it could be shown by the expression of interleukin-6 and TNFα that the FSL-1 can induce inflammation also *in vivo*. Here, in accordance to the *in vitro* data, the response to LP is much lower than that to LP*S*.

It was previously shown that bacterial LPS signals via Toll-receptor-4 (update in^29^), whereas bacterial lipoproteins and lipopeptides signal via TLR2, depending on the lipid anchor, in combination with TLR1 or TLR6^19^,^20^,^30^,^31^,^32^. Here, we could show with TLR2-transfected HEK cells, that all investigated lipopeptide compounds, LP from *N. gonorrhoeae*, from *B. burgdorferi* OspA and OspC, and from *H. influenza* induce interleukin-8 (Fig. 6). These compounds, in contrast, did not provoke IL-8 activity (except for a minor expression in the case of LP *H. influenzae*) in TLR4-transfected cells, which of course are highly stimulated by LPS. These results confirm previous results^30^ that bacterial LP products signal via TLR2 as the decisive surface receptor and that reported TLR2 activity from particular LPS such as that from *Porphyromonas gingivalis* could result from a LP contamination^32^.

The observation that only FSL-1 and MALP-2 are similarly active in the activation of human MNC as LPS is in accordance with the findings for the lipopeptides Pam_2_CSK_4_, Pam_3_CSK_4_ and lipolan, a triacylated compound consisting of the same polar head group as the former two, but with amide rather than ester linkages. The former LP were shown to be biologically active and to adopt non-lamellar aggregates corresponding to a conical conformation, whereas lipolan was biologically inactive and adopted a lamellar aggregate structure^33^. Similar arguments regarding the bioactive conical conformation of the molecules have been presented for the shortened variant of the 19 KDa LP from *M. tuberculosis*^34^. When comparing the cytokine induction caused by the different LP structures, it is striking that the diacylated lipopeptides are significantly more active than the triacylated LP, whereas the detailed amino acid sequence of the polar head groups of the LP apparently plays only a secondary role: Thus, the charge of the head groups varies from +4 (Pam_3_CSK_4_), over +1 (FSL-1), 0 (LP OspA), –1 (LP OspC, LP HiP6, MALP-2), to –2 (LP Ng H8). This indicates that (i) the general amphiphilic aggregate structure is a more decisive parameter than the detailed chemical primary structures and (ii) the lipid moiety of the lipoproteins/-peptides determines the stimulation activity as found elsewhere^10^,^19^,^20^, which is exactly the same as in the case of LPS^7^.

### Role of LP in Gram-negative species

In particular for Gram-negative bacteria, in which LPS is a membrane-building unit by forming the complete outer monolayer of the outer membrane, the role of lipoproteins/-peptides is still unclear, for example of the Braun lipoprotein linked to the murein-layer of the bacteria^35^. Generally, as shown also here, LPS is more active than LP, therefore the first line induction of inflammation in the host seems to be triggered mainly by LPS. However, there are relevant exceptions regarding the bacterial organisms. Thus, it is known that *Neisseria* frequently expresses modified LPS/ lipid A structures with high heterogeneity and less inflammation-inducing ability than other enterobacterial lipid A^36^. Here lipoprotein/peptide compounds may partially take over the role of LPS. In a similar way, *Borrelia* belongs to the small number of Gram-negative bacteria which do not possess any LPS^37^. In this bacterium the surface antigens OspA and OspC may play an important biological role as inflammation-inducing compounds (Fig. 2).

### LP as sepsis-inducing compounds

Sepsis is mainly caused by bacterial infections with an approximate identical contribution of Gram-positive and -negative species, whereas the contribution of mycoplasma and fungi - depending on the studies - lies around 5–15%^38^. Whereas for Gram-negative species the major inflammation- and sepsis-inducing compounds are endotoxins, the responsible compounds for endotoxin-free organisms (Gram-positive bacteria, fungi, mycoplasma, some Gram-negative bacteria) are poorly described or unknown. From the present data, however, it appears to be that LP are the decisive compounds for inflammation- and sepsis-induction, while LTA and PGN, which even recently were described as the responsible compounds^38^, play, if at all, only a minor role. The lipopeptide FSL-1 selected here exemplarily, showed strong inflammation in our mouse model (Table 1 and Fig. 7). High *in vivo* biological activity induced by FSL-1 was also reported by Hübschle *et al.* in rats^39^ and by Greis *et al.* in guinea pigs^40^.

### Inhibition of cytokine induction by SALP

The second purpose of the present study was to analyze the ability of a SALP, the peptide Aspidasept^®^, to suppress the cytokine production induced by the three amphiphilic compounds (LP, LTA, and PGN) in hMNC similar as it was found for LPS^24^,^25^, see Fig. 1. As shown in Figs 2, 3, 4, 5, the inhibition of the cytokine TNFα induction takes place in most cases already at a 10:1 excess weight ratio of the peptide, and at 100:1 weight ratio there is no longer any detectable cytokine expression. For FSL-1 (Fig. 7) (i) the sepsis-inducing capability of a LP leading to a high mortality in an animal model is similar to that caused by LPS and (ii) the ability of Aspidasept^®^ to provide a survival benefit could be demonstrated in the mouse model of toxemia. It should be mentioned that the galactosamine lethality model does not reproduce the course of sepsis in humans and therefore the therapeutical efficiency shown by Aspidasept^®^ in this model needs to be evaluated in more realistic animal models. However, the use of galactosamine allowed us to study *in vivo* the TNFα inducing potential of a well-characterized synthetic compound, the lipopeptide FSL-1. This is not possible in models that rely on polymicrobial infections as the causative factor of sepsis (e.g., cecal ligation and puncture). Importantly, mice used in this study for the evaluation of the FSL-1 dependent cytokine response did not receive galactosamine and therefore it is likely that the pro-inflammatory response detected in those animals may be only due to FSL-1

It should be noted furthermore, that the kind of application of FSL-1 in the mouse model (intraperitoneally) with a direct subsequent application of the SALP in one bolus does not correspond to the situation in septic patients where already a strong ‘cytokine storm’ takes place, and in which the therapeutical approach would be the continuous infusion. We think, however, that the data presented here are promising, and the retarded application of the SALP after the heavy inflammation has already started will be a task for further experiments similar as we have done it for endotoxemia^24^.

### General mechanism of LP-induced inflammation and inhibition by SALP

The peptides do not confer protection against the shortened LP variant Pam_3_CSK_4_, for which no or only a small inhibition is observed by Aspidasept^®^ (Fig. 5), nor with another inhibiting peptide Pep19–12 (not shown). To understand this discrepancy to the behaviour of lipopeptides with longer polar moiety, isothermal titration calorimetric (ITC) experiments were performed comparatively with the lipopeptides Pam_3_CSK_4_ and FSL-1 in the presence of Aspidasept^®^ (Fig. 8). The data exhibit no clear thermal reaction with the former LP (Fig. 8A), but a considerable reaction with saturation characteristics with the latter LP (Fig. 8B). These data can be understood by considering former ITC data of the LPS:peptide interaction. The inhibition of the LPS-induced cytokine secretion was interpreted as resulting from a polar (Coulomb) interaction of the N-terminal region of the peptide with the backbone region of LPS and a hydrophobic interaction of the C-terminal region with the acyl chain moiety of LPS. The data found here for the FSL-1:peptide interaction indicate a similar mechanism, leading to LP neutralization in the biological experiment by forming less immunologically active LP aggregates. Furthermore, it can be assumed that the LP have a bioactive non-lamellar aggregate structure which converts into a multilamellar organization in the presence of the peptide, similar as found for LPS^41^,^42^. We are presently investigating these questions to extend the hypothesis of such ‘generalized supramolecular conformation’, see^33^.

As reason for the lacking interaction of Pam_3_CSK_4_ with the peptide, which does not lead to an inhibition of the cytokine induction, the polar backbone of this LP should be considered. It has only the short amino acid sequence SKKKK with four positive charges, which therefore prevents a strong Coulomb interaction with the positively charged N-terminal region of the peptide. Regarding the biological relevance, it should be noticed that Pam_3_CSK_4_ is a synthetic shortened variant of naturally occurring lipoproteins, and is not a constituent component in bacterial membranes.

When adding the polycationic polymyxin B (PMB), a traditional AMP^43^ to the shortened 19 KDa LP, a similar lack of interaction was observed^34^. In the latter publication it was found that the non-lamellar cubic structure of LP 19 KDa did not change upon interaction with PMB in accordance with a lack of biological inhibition of the stimulation of macrophages.

Former publications described LTA and PGN as inflammation-inducing components of Gram-positive bacteria (reviews in^11^ and^38^). Other authors have found that these compounds in purified or synthetic form did not show any activity or only at high concentrations (>10 μg/ml)^15^, (see also reviews by Nakayama *et al.*^9^ and by Zähringer *et al.*^13^). Our own data indicate that commercial LTA from *B. subtilis* exhibits some activity, whereas the signal from *S. aureus* LTA is only marginal (Fig. S3 and^27^), which might be a hint that the activity from the former LTA actually could be due to a contamination. The observation of a minor activity is also valid for PGN (Fig. 4). However, in this context the more important observation is, that the SALP are able to inhibit any cytokine production, whether induced by LTA or by a LP contaminant and are thus able to represent a general neutralizing agent of infection-inducing Gram-negative as well as Gram-positive amphiphiles.

Finally, the question with respect to the physiological importance of the findings reported here may arise. In the case of endotoxins, LPS concentrations of as low as 1 ng/ml may be sufficient to induce sepsis in humans^44^. Possible LP concentrations in septic patients induced by LPS-lacking bacteria are unknown. But for various of the LP investigated here, concentrations in the range of 10 to 100 ng/ml were sufficient to induce cytokines, which might be easily reached by a sufficiently high number of bacteria in human blood.

## Methods

### Bacterial cell wall compounds

LPS from *Salmonella enterica* Minnesota rough mutant R60 was extracted from log-phase bacteria growing in LB medium (at 37 °C) by the phenol:chloroform:petrol ether (PCP) method, purified, and used in the natural salt form^45^. The purity was examined by MALDI-TOF mass spectrometry and only LPS samples with the predominant *Salmonella* lipid A chemical species (a hexa-acylated diglucosamine backbone phosphorylated at positions 1 and 4′ and bearing acyl chains in amide and ester-linkage at positions 2 and 2′, and 3 and 3′, respectively), were used for subsequent assays.

LTAs were purchased from Sigma-Chemie (Deisenhofen, Germany), one type from *Staphylococcus aureus* (L-2515) and another from *Bacillus subtilis* (L-3265) and used without further purification. LTA from *S. aureus* SA113Δ*lgt* was prepared as described elsewhere^15^.

The LP (SitC) from *Staphylococcus aureus* was overexpressed in a his-tagged form from *S. aureus* SA113 (pTX30SitC-his) and purified as described by Müller *et al.*^46^. *S. aureus* was reported to express either diacylated or triacylated LP depending on the environmental conditions^9^. Asanuma *et al.*^8^, however, showed that in *S. aureus* the LP occur mainly in the *N*-acylated triacyl form.

For the isolation of PGN from *Staphylococcus aureus* strain SA113Δ*lgt*^14^, kindly provided by F. Götz (Tübingen, Germany)), a mutant strain that is deficient in acylation of its prelipoproteins, was grown as an overnight culture in Todd Hewitt Medium at 37 °C and stirring at 140 rpm (until an OD_660_ of 1.7, approximately). For killing of bacteria, the culture was boiled for 30 min and then allowed to cool down to room temperature. Cells were collected by centrifugation (9,000 rpm, 20 min, 4 °C), and washed three times with water, three times with acetone and again with water. The resulting pellet was freeze-dried. PGN was than prepared following the protocol of de Jonge *et al.*^47^, including treatment with α-amylase, DNase, RNase, trypsin, alkaline phosphatase and hydrofluoric acid.

### Lipopeptide (LP) synthesis

Various LP were synthesized by EMC microcollections GmbH (Tübingen, Germany). The synthesis of LP was carried out by fully automated solid phase peptide synthesis and Fmoc/tBu chemistry on TCP resin. For coupling of amino acids a seven-fold molar excess of single Fmoc-L-amino acids was used. The peptide resins carrying the sequences were elongated with the unusual lipoamino acid Pam_3_Cys-OH. Pam_2_. The LP FSL-1 and MALP-2 were obtained by elongation of the peptide with the unusual amino acid Fmoc-Dhc (Dhc: (S)-2,3-dihydroxy-2-(R,S)-propyl-(R)-cysteine) followed by O-palmitoylation and Fmoc deprotection. The couplings were carried out in DMF:CH_2_Cl_2_ (1:1) with DIC/HOBt in threefold excess within 3 h and were monitored by Kaiser assay. The LP were cleaved off the resin by treatment of the resins with trifluoroacetic acid/phenol/ethanedithiol/thioanisole (96:2:1:1) and purified by precipitation. Analytical characterisation of all LP was done by electrospray mass spectrometry (ESI-MS).

Specifically, the chemical structures of the LP are as follows:

### Synthetic di- and triacyl LP homologous to the N-terminal part of bacterial LP:

Pam_3_CSK_4_: ‘Water-soluble’ analogue of natural LP with modified sequence.

Pam_3_Cys-SKKKK.

LP OspA: A shortened analogue of OspA from *Borrelia burgdorferi*

Pam_3_Cys-KQNVSSLDEKNSVSV.

LP Osp: A shortened analogue of OspC from *Borrelia burgdorferi*

Pam_3_Cys-NNSGKDGNTSANSAD.

LP P6: A shortened analogue of protein P6 from *Haemophilus influenzae*.

Pam_3_Cys-SSSNNDAAGNGAAQT.

LP H8: A shortened analogue of LP blp NG from *Neisseria gonorrhoeae.*

Pam_3_Cys-SQEPAAPAAEATPAG.

### Synthetic diacyl LP:

FSL-1: A shortened analogue of the 44-kDa LP LP44 of Mycoplasma salivarium.

Pam_2_Cys-GDPKHPKSF.

MALP-2: A shortened analogue of macrophage-activating LP from *Mycoplasma fermentans*.

Pam_2_Cys-GNNDESNISFKEK.

Details of these compounds can be found on the homepage of EMC-Microcollections (Tübingen, Germany). (http://www.microcollections.de/).

### Synthetic anti-LPS peptides (SALP)

The synthesis and purification of the Pep19-2.5 (Aspidasept^®^) was described previously^25^, the batches used here were produced by BACHEM (Bubendorf, Switzerland). The amino acid sequence of this 20′mer is GCKKYRRFRWKFKGKFWFWG. The peptides Pep19–4 (GKKYRRFRWKFKGKWFWFG), Pep9-8 (GRRYKKFRWKFKGRWFWFG) and Pep19–12 (GCRRFKKFKKWRYRGRFWFWCFG) were synthesised in the Research Center Borstel as described earlier^26^.

All peptides were amidated at the C-terminal end and had a purity of >95% as measured by HPLC and MALDI-TOF mass spectrometry.

### Stimulation of MNC cells and macrophages by LPS and LP

The stimulation of MNC was performed as described previously^24^,^25^. Briefly, MNC were isolated from heparinized blood of healthy donors by the Hypaque-Ficoll density gradient method. The cell number was equilibrated at 5 × 10^6^ cells/ml RPMI 1640 containing 2  mM L-glutamine, 100 U/ml penicillin and 100 μg/ml streptomycin. For stimulation, 200 μl/well MNC were transferred into 96-well culture plates. The stimuli were serially diluted in RPMI 1640 and added to the cultures at 20 μl per well. The cultures were incubated for 4 h at 37 °C under 5% CO_2_. Cell-free supernatants were collected after centrifugation of the culture plates for 10 min at 400 × g and stored at −20 °C until the determination of the cytokine content. Immunological determination of TNFα in the cell supernatant was performed in a sandwich-ELISA as described earlier (OptEIA; BD, Heidelberg, Germany). TNFα was determined and measured following manufacturer’s instructions and results are the average of two assays.

In some stimulation experiments, also macrophages were used. Briefly, MNC were isolated from peripheral blood from healthy donors by the Hypaque-Ficoll gradient method. To differentiate the monocytes to macrophages cells were cultivated in Teflon bags in the presence of 2 ng/ml M-CSF in RPMI 1640 medium (endotoxin <0.01 EU/ml; Biochrom, Berlin, Germany) containing 100 U/ml penicillin, 100 μg/ml streptomycin, 2 mM L-glutamine and 4% heat-inactivated human serum type AB at 37 °C and 6% CO_2_. On day 6, cells were washed with PBS, detached by Trypsin-EDTA treatment and seeded at 1 × 10^6^ cells/ml in complete medium in 96-well tissue culture dishes (Nunc, Wiesbaden, Germany). After stimulation of cells with various LP for 4 h, cell-free supernatants of duplicate samples were collected, pooled and stored at −20 °C until determination of cytokine content. Data shown are representative of at least three independent experiments.

LP preparations were pre-warmed for 30 min at 37 °C, and added to the cultures at 20 μl per well. In inhibition experiments, different SALP were added directly after LPS/LP/LTA administration.

Since the data of the stimulation of human MNC by the bacterial and synthetic LP was taken over a time period of more than 6 years, the TNFα production lead to considerable variations due to different batches of LP and of Pep19-2.5, and in particular due to variations resulting from blood samples of different donors. Because the main emphasis laid here is the efficiency of the peptide to inhibit the cytokine production, most figures describe the inhibition efficieny of the peptide in %, by presenting the means ± SEM of at least 3 independent experiments. The absolute values of the cytokine production, where of note, is given separately in the text.

### Transient Transfection and Stimulation of HEK293 Cells

For HEK cell activation, human IL-8 was determined by sandwich enzyme-linked immunosorbent assay using IL-8 cytoset from BIOSOURCE (Life Technologies GmbH, Darmstadt Germany) exactly according to the manufacturer’s protocol. For transient transfection, HEK293 cells were plated at a density of 2.5 × 10^4^/well in 96-well plates in Dulbecco’s modified Eagle’s medium (PAA, Pasching, Germany). The following day, cells were transfected using Lipofectamine (Life technologies) according to the manufacturer’s protocol. Expression plasmid containing the FLAG-tagged version of human TLR2 was a kind gift from P. Nelson, Seattle, WA and subcloned into pREP9 (Life Technologies). TLR2 plasmid was used at 100 ng/transfection. After 24 hours of transfection, cells were washed and stimulated for 18 hours with indicated compounds and positive controls. Finally, supernatants were collected and the interleukin-8 content was quantified using a commercial ELISA (Life technologies).

Data shown are the mean ± S.D. of triplicate samples of one experiment and representative of two independent experiments.

### Mouse model of cytokine induction and toxemia.

Seven-week-old female SPF-free Balb/C mice of 20 g of body weight, approximately, were purchased from Harlan Spain (Harlan Interfauna Iberica S.A., Barcelona, Spain) and randomly distributed in experimental groups (at least 4 animals per group except for the determination of lethal dose (n = 3)). Sample size was calculated based on previous results with the same animal model^24^. Animals were housed in individually ventilated cages (5 mice per cage) bedded with hardwood bedding. Mice were provided ad libitum access to feed and water and lighting was adjusted to a 14-hour light/10-hour dark cycle. During the experiments, the condition of the animals was monitored daily every 4 hours and animals displaying persistent motor ataxia and hunched posture were euthanized. For this purpose, mice were sacrificed by cervical dislocation by a technician with a demonstrated high degree of technical proficiency.

To study if FSL-1 had pro-inflammatory cytokine-inducing activity, animals were intraperitoneally inoculated with 40 μg of FSL-1 resuspended in 200 μl of pyrogen-free saline. Blood samples were taken both at 1.5 h or 4 h after FSL-1 challenge to measure TNFα and IL-6 levels. For this purpose, mice were anesthetised, a blood sample was obtained by the retroorbital plexus and serum cytokine levels were measured using Quantikine Immunoassays kits (R&D systems, Minneapolis, USA) following manufacturer’s instructions. Differences between groups were statistically analyzed using the Mann-Whitney U test.

To evaluate the ability of Aspidasept^®^ to neutralize the FSL-1 induced pro-inflammatory cytokines, a group of animals received by the intraperitoneal route first 40 μg of FSL-1 and then 400 μg of the drug resuspended in 100 μl of pyrogen-free saline. This second injection was administered immediately after FSL-1 challenge at a different site of the peritoneum. TNFα and IL-6 levels were measured (see above) at 1.5 h or 4 h after challenge, respectively, coinciding with the peak of cytokine secretion, as determined in preliminary experiments.

To determine the lethal dose of FSL-1 in mice, the method of Galanos and collaborators^48^ was followed with some modifications. Briefly, animals were intraperitoneally inoculated with increasing amounts of FSL-1 (50 ng, 500 ng, 5 μg and 50 μg) supplemented with 18 mg of D-galactosamine in a final volume of 200 μl of pyrogen-free saline. Galactosamine is known to sensitize mice to pro-inflammatory compounds such as LPS, thus making it possible to use lower amounts of the stimulatory drug^46^. The ability of Aspidasept^®^ to protect mice from FSL-1 dependent lethal toxemia was assessed by co-administering FSL-1 (4 μg/mouse) with D-galactosamine (18 mg/mouse) intraperitoneally. Aspidasept^®^ (200 μg/mouse) was administered immediately after FSL-1 at a different site of the peritoneum and the physical activity of each animal was rated every 4 hours according to the following criteria: 1 = very active; 2 = active; 3 = less active; 4 = slow; 5 = lethargic; † = dead.

Results were globally analyzed by means of a Kaplan-Meier survival analysis (SPSS 15.0). When the survival plots were parallel, the data were compared by the log-rank test, whereas the Breslow-Gehan-Wilcoxon test was applied when the plots intersected.

Procedures involving animals were conducted in accordance with the European and Spanish regulations (Directive 2010/63/EU, Recommendation 2007/526/EC, Real Decreto 53/2013 and Ley 32/2007) and were approved by the Animal Research Committee of the University of Navarra (protocol No. E50-12(069-09 E4)[A]).

### Isothermal Titration Calorimetry (ITC)

Microcalorimetric measurements of peptide binding to selected LP, i.e., Pam_3_CSK_4_ and FSL-1 were performed on a MCS isothermal titration calorimeter (Microcal, Freiburg, Germany) at 37 °C, as previously described^24^,^25^. The LP containing solutions (0.05 mM, prepared as described above) were dispensed into the microcalorimetric cell (volume 1.5 ml), and the peptide solutions (1 mM) were loaded into the syringe compartment (volume 0.1 ml). After temperature equilibration, the peptide was titrated in 3 μl aliquots every 5 min into the LP-containing cell under constant stirring and the heat of interaction after each injection measured by the MCS instrument was plotted versus time.

### Förster Resonance Energy Transfer Spectroscopy (FRET)

The ability of selected peptides to intercalate into LP aggregates was investigated exemplarily for FSL-1 and for LP SitC by FRET as described earlier^26^. Briefly, the LP samples were doubly labelled with the fluorescent phospholipid dyes N-(7-nitrobenz-2-oxa-1,3-diazol-4-yl)-phosphatidyl ethanolamine (NBD-PE) and N-(lissamine rhodamine B sulfonyl)-phosphatidylethanolamine (Rh-PE) (Molecular Probes, Life Technologies). Intercalation of unlabeled molecules into the doubly labeled liposomes leads to probe dilution and to a lower FRET efficiency: Therefore, the emission intensity of the donor I_D_ increases and that of the acceptor I_A_ decreases (for the sake of clarity, only the quotient of the donor and acceptor emission intensity will be shown here).

In all the experiments, the peptides (100 μl of a 100 μM Hepes solution) were added to doubly labelled LP (900 μl of a 10 μM solution) at 50 s after equilibration. NBD-PE was excited at 470 nm and the donor and acceptor fluorescence intensities were monitored at 531 and 593 nm, respectively, and the fluorescence signal I_D_/I_A_ was recorded for further 250 s. The measurement was repeated twice.

## Additional Information

**How to cite this article**: de Tejada, G. M. *et al.* Lipoproteins/peptides are sepsis-inducing toxins from bacteria that can be neutralized by synthetic anti-endotoxin peptides. *Sci. Rep.* **5**, 14292; doi: 10.1038/srep14292 (2015).

## Figures and Tables

**Table 1:** Survival and physical activity of Balb/C mice at different time points after inoculation of increasing doses of FSL-1.

Amount of FSL-1	4 hours	8 hours	24 hours	48 hours
50 ng	1 2 2	1 1 2	1 1 2	1 1 2
500 ng	3 3 3	3 3 †	† † †	† † †
5 μg	4 4 4	5 5 4	† † †	† † †
50 μg	5 5 5	4 † †	† † †	† † †

Groups of animals (n = 3) were intraperitoneally inoculated with the indicated amount of FSL-1 supplemented with D-Galactosamine (18 mg/mouse) in pyrogen-free sterile saline and at the indicated time-point the physical activity of each animal was rated according to the following criteria: 1 = very active; 2 = active; 3 = less active; 4 = slow; 5 = lethargic; † = dead.
